# Mapping the Interplay Between Childhood Trauma, Substance Use, and Psychopathology in Early Psychosis: A Network Analysis Approach

**DOI:** 10.1093/schbul/sbaf253

**Published:** 2026-03-21

**Authors:** Dimitrios Kiakos, Luis Alameda, Lilith Abrahamyan-Empson, Carolina Spanevello, Marianna Gorgellino, Livia Alerci, Nadir Mebdouhi, Philippe Conus, Sandra Vieira

**Affiliations:** Service of General Psychiatry, Lausanne University Hospital (CHUV), Lausanne, 1003, Switzerland; Service of General Psychiatry, Lausanne University Hospital (CHUV), Lausanne, 1003, Switzerland; Department of Psychosis Studies, Institute of Psychiatry, Psychology and Neuroscience, King's College, London, SE5 8AB, United Kingdom; National Psychosis Unit, South London and Maudsley NHS Foundation Trust, London, SE5 8AZ, United Kingdom; Service of General Psychiatry, Lausanne University Hospital (CHUV), Lausanne, 1003, Switzerland; Service of General Psychiatry, Lausanne University Hospital (CHUV), Lausanne, 1003, Switzerland; Service of General Psychiatry, Lausanne University Hospital (CHUV), Lausanne, 1003, Switzerland; Service of General Psychiatry, Lausanne University Hospital (CHUV), Lausanne, 1003, Switzerland; Service of General Psychiatry, Lausanne University Hospital (CHUV), Lausanne, 1003, Switzerland; Service of General Psychiatry, Lausanne University Hospital (CHUV), Lausanne, 1003, Switzerland; Service of General Psychiatry, Lausanne University Hospital (CHUV), Lausanne, 1003, Switzerland; Department of Psychosis Studies, Institute of Psychiatry, Psychology and Neuroscience, King's College, London, SE5 8AB, United Kingdom

**Keywords:** cannabis and psychosis, trauma-related pathways, symptom networks, depressive symptoms, psychosis interventions and prevention

## Abstract

**Background and Hypothesis:**

Evidence suggests a complex interplay among childhood trauma (CT), substance use, and psychopathology in psychosis, yet the precise nature of these relationships remains unclear. Network analysis offers distinct advantages for examining these factors simultaneously, as it can capture multidirectional associations across multiple trauma subtypes, substances, and symptoms that conventional methods may overlook.

**Study Design:**

In this study, data from 317 patients with early psychosis were used. CT was assessed across 5 subtypes (sexual, physical, and emotional abuse; physical and emotional neglect) using a tailored questionnaire completed by case managers. Current use of alcohol, opioids, cocaine, cannabis, and amphetamines was evaluated with the Case Manager Rating Scale for Substance Abuse. Symptoms were measured with the Positive and Negative Syndrome Scale. A mixed graphical model was fitted to estimate conditional associations among CT, substance use, and psychopathology. Further analyses on the network’s connectivity were conducted.

**Study Results:**

Three distinct patterns of connection emerged, respectively associated with cannabis use, depressive symptoms, and attentional difficulties, linking trauma to psychopathology. Cannabis emerged as a central bridge, linking trauma to both symptom severity and broader substance use.

**Conclusions:**

These findings highlight the nuanced relationships between CT, substance use, and psychopathology. They further suggest that cannabis may serve both as a catalyst for psychopathology and as a gateway to broader substance use among trauma-exposed individuals. Together, these findings may support more comprehensive clinical formulation and informed treatment planning in early intervention settings.

## Introduction

There is considerable evidence that exposure to childhood trauma (CT)—including sexual abuse, physical abuse, emotional abuse, and neglect—is associated with an increased risk of developing psychotic disorders (PD) and, among those with PD, with greater symptom severity and a longer illness course.^1^ At the same time, CT is a known risk factor for substance use disorder (SUD).^2^^,^^3^ Furthermore, SUD in the context of PD has been associated with worse clinical outcomes.^4^^,^^5^

Nevertheless, despite significant evidence pointing to a triangular relationship between CT, SUD, and clinical outcomes in PD, the exact nature of this relationship remains understudied. The few existing studies have focused exclusively on cannabis use and have yielded inconsistent results.

Studies in the general population^6^^,^^7^ have shown that when both CT and cannabis use are present, their impact on psychotic symptoms is multiplicative. However, studies in early psychosis samples^8^^,^^9^ testing this hypothesis did not find such an interaction. General population studies have investigated whether cannabis use mediates the relationship between CT and psychotic symptoms. Van Nierop et al.^10^ found no evidence of mediation, whereas other studies reported that cannabis use partially mediates the association between CT and hallucinations^11^ and between CT and paranoia.^12^

The current literature exploring the relationship between CT, SUD, and psychotic symptoms has several notable limitations. First, substances other than cannabis that have also been linked to CT^2^^,^^3^ and clinical outcomes in PD^4^ are generally overlooked. Second, most studies define cannabis use merely as having used it at least once in a lifetime, without establishing a clear temporal link between use and the onset of psychotic symptoms. Finally, the contradictory results may partly stem from limitations inherent to the analytical methods employed in these studies.

In recent years, network analysis has emerged as a promising method in psychiatric research. It is a data-driven approach that conceptualizes symptoms, along with psychological, biological, and social components of disease, as mutually interacting and often reciprocally reinforcing elements of a complex network.^13^ Network analysis offers several advantages over traditional statistical methods, which may be particularly relevant for studying the interactions among CT, SUD, and clinical symptoms.

First, it allows for the simultaneous analysis of multiple variables and enables examination of clinical constructs at the item level. This is especially important when studying CT in PD, as different trauma subtypes exhibit distinct patterns of association with clinical symptoms.^14^ Several studies^15–17^ using network analysis to explore links between CT, symptoms, and other clinical outcomes in PD have demonstrated the high granularity of CT-related and illness-related constructs, as well as complex patterns of association among them. Second, network analysis does not require prior assumptions about the direction of causality, which is particularly important given evidence that the relationship between cannabis use and psychotic symptoms may be bidirectional.^18^

To our knowledge, the study by Bosma et al.^19^ is the only one to date that has employed network analysis to explore the interactions between CT, cannabis use, and clinical symptoms in PD. The study examined data from individuals with non-affective psychotic disorders and their healthy siblings, and found that in both groups, cannabis use was not directly connected to CT and had only a limited influence within the network. However, the study relied on total scores for trauma and symptom dimensions, likely failing to capture the more nuanced relationships between specific CT subtypes and other elements within the network.

The present study aims to address these gaps in the literature by examining the relationships among CT, substance use, and clinical symptoms in PD using an item-level network analysis approach. Specifically, the study seeks to (1) characterize patterns of association between prevalent CT subtypes, current use of commonly used substances, and clinical symptoms in a sample of early psychosis patients, and (2) identify the environmental exposures and symptoms that occupy influential positions within the network and may therefore warrant particular attention in assessment and clinical care planning within early intervention settings.

## Methods

### Participants and Procedure

This study utilized data from patients enrolled in the Treatment and Early Intervention in Psychosis Program (TIPP), an early intervention service within the Department of Psychiatry at Lausanne University Hospital in Switzerland.

The program’s inclusion criteria^20^ are as follows: (1) age between 18 and 35; (2) having crossed the psychosis threshold as defined by the “Psychosis threshold” subscale of the Comprehensive Assessment of At-Risk Mental States Scale^21^; (3) residing in the catchment area (Lausanne and surroundings; population about 300 000). Exclusion criteria include: (1) prior use of antipsychotic medication for more than 6 months; (2) psychosis related to acute intoxication or organic brain disease; (3) an intelligence quotient below 70.

For some individuals, TIPP represents their first contact with psychiatric services for psychotic symptoms. Others are referred from community clinics or hospitals where treatment has already been initiated. For patients who are partially or fully remitted at the time of evaluation, symptom severity at its peak is retrospectively assessed using information from the patient, healthcare professionals, and medical records. The decision regarding whether a patient has crossed the psychosis threshold is made during a multidisciplinary team meeting involving senior clinical staff.

Each patient is followed for a duration of 3 years and receives an integrated, biopsychosocial treatment plan. This includes psychiatric care, psychotherapy, psychoeducation, family support, cognitive assessment and remediation, social and vocational assistance, substance use interventions, and pharmacological treatment.

Upon enrollment, a structured questionnaire is completed by each patient’s case manager. This tool gathers information on demographic characteristics, medical history, exposure to life events, symptom profile, and functioning. Data are collected from both patients and their families during the initial weeks of care and may be updated over time as new information becomes available. Follow-up assessments are conducted regularly throughout the 3-year follow-up period. During these assessments, psychopathology is evaluated by a trained psychologist, while the case manager assesses other clinical domains, including functioning, insight, treatment adherence, substance use, trauma exposure, suicide attempts, and forensic history.

Access to TIPP clinical data for research is granted under the approval of the Human Research Ethics Committee of the Canton of Vaud (CER-VD; protocol #2020-00272). Upon enrollment, patients are routinely informed about the potential use of their clinical data for research, and consent is obtained accordingly. The refusal rate is low, at approximately 4%.

### Measures and Data Collection

#### Childhood Trauma

CT was assessed using a tailored questionnaire that evaluates the presence of 12 distinct trauma types and records the age at which each occurred. Data on trauma exposure were collected by trained case managers through a comprehensive and iterative assessment process. Rather than relying solely on a single interview, scale, or self-report, the evaluation was conducted within the context of a trusting relationship established progressively throughout the treatment period. Case managers met regularly with patients, allowing for the gradual development of trust and a deeper understanding of their personal histories. With the patient’s consent—and when clinically appropriate—additional information was obtained from family members to corroborate and enrich the trauma history. To ensure data reliability, patients were excluded from the present analysis in cases where discrepancies in reporting or uncertainty regarding the nature or timing of traumatic events were identified.

Five types of CT were included in this study: sexual abuse, emotional abuse, physical abuse, emotional neglect, and physical neglect. These categories represent the most commonly reported forms of trauma during childhood.^22^ Sexual abuse refers to sexual molestation and/or rape. Physical abuse includes physical attack or assault, or being repetitively beaten by parents, relatives, or caregivers. Emotional abuse is defined as verbal assaults on a child’s sense of worth or well-being, or any humiliating or demeaning behavior directed toward a child by an adult or older person. Emotional neglect is defined as the failure of caretakers to meet child’s basic emotional and psychological needs, including love, belonging, nurturance, and support. Physical neglect involves the failure of caretakers to meet a child’s basic physical needs, including food, shelter, clothing, safety, and health care.

Only traumatic events that occurred before the age of 16 were included in the analysis, as trauma experienced after this age may overlap with the prodromal phase of a first psychotic episode.^23^

#### Substance Use

Substance use over the past month was assessed by case managers using the Case Manager Rating Scale (CMRS) for Substance Abuse, a 5-point scale originally developed as a research tool for clinical case managers to evaluate the severity of substance-related problems among patients with severe psychiatric disorders in the community.^24^^,^^25^ The CMRS has demonstrated high interrater reliability and strong concurrent validity.^24^

A rating of (1) (Absent) indicates no substance use during the assessed period. (2) (Mild) reflects occasional, recreational, or minimal use that was not judged clinically problematic. (3) (Moderate) corresponds to persistent substance use associated with clear and ongoing psychosocial or physical impairments. (4) (Severe) and (5) (Extremely Severe) indicate regular, excessive use, with severity differentiated by the extent and impact of associated problems. The full scale is provided in the Supplementary Note 1.

For this study, 5 substances were included in the analysis—alcohol, opioids, cocaine, cannabis, and amphetamines—as they represent the most commonly used substances among patients with PD.^26^

#### Clinical Symptoms

Clinical symptoms were evaluated by a trained psychologist using the Positive and Negative Syndrome Scale (PANSS).^27^ The PANSS is the most widely used instrument for assessing symptom severity in individuals with PD and has demonstrated sufficient reliability and construct validity.^28^ The scale consists of 30 items grouped into 3 subscales: positive symptoms, negative symptoms, and general psychopathology. Each item is rated on a 7-point Likert scale, ranging from 1 (absent) to 7 (extremely severe). In this study, the PANSS showed strong internal consistency, with a Cronbach’s alpha of 0.87.

### Study’s Design

This study employed a cross-sectional design, focusing on patients’ initial comprehensive assessment within TIPP. For the majority of participants, this assessment took place approximately 2 months after enrollment in the program. However, for a subset of patients who remained highly symptomatic and unable to complete the evaluation at that time, the assessment was postponed until 6 months after treatment initiation. In all cases, data on substance use and symptom severity were obtained concurrently, either at the 2- or 6-month mark, depending on each patient’s clinical stability.

### Statistical Analysis

Analyses were conducted at the item level to capture fine-grained relationships that might be obscured by composite scores and to reflect the full complexity of interactions among the studied constructs without imposing restrictive assumptions. Data for 5 types of CT (sexual abuse, physical abuse, emotional abuse, emotional neglect, and physical neglect), the CMRS scores for 5 substances (alcohol, opioids, cocaine, cannabis, and amphetamines), and the 30 items of the PANSS were used to estimate a network.

Since the dataset included both ordinal variables (CMRS and PANSS scores) and binary variables (CT), a Mixed Graphical Model (MGM) was applied, which is specifically designed for handling mixed data. In MGMs, the strength of associations between variables is quantified through regression coefficients derived from generalized linear models of standardized variables.^29^ Missing data (1.5% of the dataset) were assumed to be missing at random and were imputed using the k-nearest neighbors method,^30^ with *k* set to 5. Consistent with recent studies^16^^,^^31^^,^^32^ ordinal variables were treated as continuous, and to address skewness in the data, a nonparanormal transformation^33^ was applied prior to network estimation.

For model selection, L1-regularized regression (LASSO) was employed. LASSO introduces a penalty parameter that shrinks weak associations between variables toward zero, effectively eliminating those likely to reflect sampling variability rather than true relationships in the studied population.^30^ This approach to controlling false positives is preferred in network analysis, as it offers several advantages over traditional significance testing—namely, it avoids arbitrary significance thresholds and the loss of power associated with multiple-testing corrections,^34^ while providing a principled means of identifying robust and replicable associations among variables.

In psychometric network modeling, 2 methods are commonly used to determine the optimal penalty parameter: the Extended Bayesian Information Criterion (EBIC) and cross-validation (CV). A large-scale simulation study^35^ showed that while EBIC favors precision, CV tends to offer greater sensitivity, especially when estimating MGMs with smaller samples. As this study was exploratory in nature and prioritized discovery over strict hypothesis testing, CV was selected, using 10 folds. However, given the limited empirical knowledge regarding the comparative performance of regularized estimators in network analysis, a secondary analysis was also conducted using EBIC (with γ = 0.25) to assess whether the main findings remained robust under a more conservative regularization approach.

The network was visualized using a node-and-edge structure, where nodes represent variables and edges connecting node pairs indicate associations after controlling for all other variables in the network. Edge weights, which quantify the strength of conditional associations between variables, were computed. These weights are reflective of the regression coefficients from a multiple regression model and can therefore be interpreted as how well one variable helps explain variation in another. In this framework, 2 connected nodes mutually predict each other, and any intermediary node between them may act as a potential mediator of this predictive relationship.^15^

Subsequent analyses examined both centrality and bridge centrality within the network. Centrality metrics help identify nodes that play a key role in the flow of information across the network, such that their deactivation could meaningfully influence overall network dynamics. With appropriate theoretical justification and empirical validation, these nodes may serve as potential targets for intervention.

Node centrality, which reflects the relative importance of a node in facilitating information transmission within the network,^36^ was assessed by computing node strength, as it is considered the most stable and interpretable among centrality measures.^37^ Node strength is defined as the sum of the absolute edge weights connected to a node, indicating the overall strength of its associations with other nodes in the network.^36^ To identify key nodes linking CT with substance use and clinical symptoms, bridge strength was also computed. Bridge strength is defined as the sum of edge weights connecting a given node to nodes in other predefined communities,^38^ thereby allowing the identification of nodes that function as intermediaries across distinct domains. In this study, 3 communities were defined: CT, substance use, and clinical symptoms.

Beyond connectivity, node predictability was examined to assess how much of a node’s variance is explained by its directly connected neighbors in the network. High predictability suggests that the node is strongly influenced by other nodes within the network, whereas low predictability indicates that its variance is largely influenced by factors outside the network.^39^

To evaluate the stability and accuracy of the estimated network, several bootstrapping procedures were conducted.^37^

Edge weight stability was assessed using non-parametric bootstrapped confidence intervals (CIs). This procedure quantifies how consistently the same connections would emerge if many slightly different samples were drawn from the same population. In practice, it involves repeatedly resampling the dataset with replacement and re-estimating the network to generate a sampling distribution for each edge weight. Narrower CIs indicate greater stability of the estimated edge strengths, suggesting that the observed connections are unlikely to be due to sampling variability.

Node-strength accuracy was evaluated using a case-drop bootstrap procedure, which examines whether the most influential nodes remain stable when the network is re-estimated on slightly altered datasets. In this procedure, centrality indices are recalculated after randomly removing increasing proportions of cases. This allows assessment of how sensitive node strength estimates are to fluctuations in the data. From these analyses, the correlation stability (CS) coefficient was computed, representing the maximum proportion of cases that can be dropped while maintaining a correlation of at least 0.7 with the original centrality estimates. A CS coefficient above 0.25 is considered acceptable.

All analyses were conducted using R statistical software (version 4.4.1).^40^ The mgm package^29^ was used for network estimation and predictability computation, the qgraph package^41^ was used for network visualization, and the bootnet package^37^ was employed for stability and accuracy analyses.

## Results

This study included a total of 317 patients, of whom 225 were assessed at 2 months and 92 at 6 months following treatment initiation. The sample consisted of 109 women (34.38%) and 208 men (65.62%), with a mean age of 24.87 years. The mean duration of untreated psychosis was 429.82 days. Regarding treatment, 172 patients (54.25%) were not receiving any antipsychotic medication. Among those on medication, 94 patients (29.65%) were partially compliant and 51 (16.08%) were fully compliant with their treatment.

A total of 163 patients (51.42%) in the sample reported substance use, with 54 of them (17.03%) using more than one substance. Specifically, 129 patients (40.69%) reported alcohol use, 81 (25.55%) used cannabis, 8 (2.52%) used cocaine, 6 (1.89%) used opioids, and 3 (0.95%) used amphetamines.

Patients’ demographic and clinical characteristics are summarized in Table 1.

**Table 1:** Demographic and Clinical Characteristics of the Study Sample

**Variables**	**Study sample (*n* = 317)**
**Gender, *n* (%)**	
Female	109 (34.38%)
Male	208 (65.62%)
Age (years), Mean (SD)	24.87 (4.71)
**Marital status, *n* (%)**	
Single	278 (87.69)
Married	22 (6.94)
Divorced	10 (3.15)
Cohabitation	7 (2.20)
**Completed post-school training, *n* (%)**	
None	25 (7.88)
Apprenticeship	237 (74.76)
Vocational school	20 (6.30)
University	35 (11.04)
Duration of untreated psychosis (days), Mean (SD)	429.82 (802.76)
**Medication, *n* (%)**	
No medication	172 (54.25)
Partial medication	94 (29.65)
Complete medication	51 (16.08)
**PANSS, Mean (SD)**	
PANSS positive subscale	1.90 (1.36)
PANSS negative subscale	2 (1.38)
PANSS general psychopathology subscale	1.76 (1.26)
PANSS total score	1.85 (1.31)
**CMRS, Mean (SD)**	
Alcohol	1.47 (0.64)
Opioids	1.04 (0.34)
Cocaine	1.03 (0.16)
Cannabis	1.38 (0.77)
Amphetamines	1.01 (0.10)
**Childhood trauma, *n* (%)**	
Sexual abuse	40 (12.61)
Physical abuse	61 (19.24)
Emotional abuse	19 (5.99)
Emotional neglect	36 (11.35)
Physical neglect	18 (5.67)

Abbreviations: CMRS = Case Manager Rating Scale for Substance Abuse; PANSS = Positive and Negative Syndrome Scale.

### Network Structure

**Figure 1:**
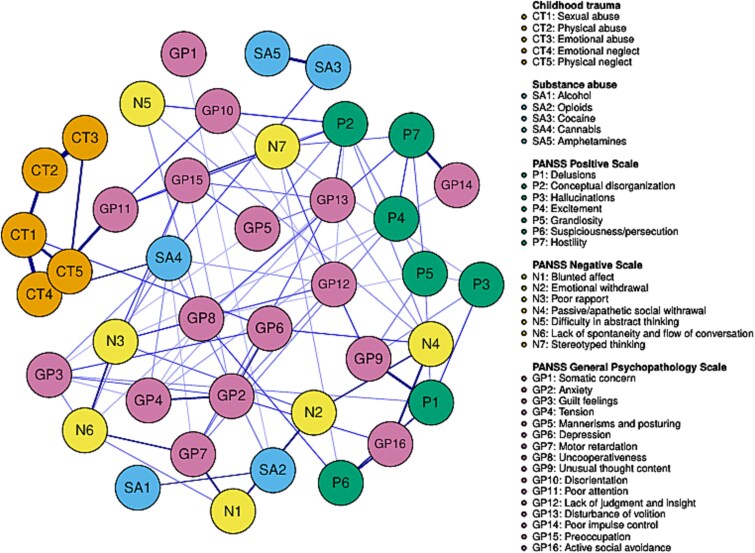
Partial Correlation Network Among the 5 Childhood Trauma Domains (Sexual Abuse, Physical Abuse, Emotional Abuse, Emotional Neglect, and Physical Neglect), the 5 Substances (Alcohol, Opioids, Cocaine, Cannabis, and Amphetamines), and all Individual Symptoms from the 3 PANSS Sub-scales (Positive, Negative, and General Psychopathology).

The estimated network is presented in Figure 1. It comprised 93 edges. All edges and their corresponding weights are detailed in the Table S1. The network was well-connected, with no isolated nodes.

The 30 PANSS nodes formed a dense complex, indicating that these symptoms frequently co-occur. The CT nodes formed a tightly interrelated cluster located on the periphery of the PANSS complex, suggesting strong internal associations among trauma types but weaker direct links to symptom nodes. The substance abuse nodes were more dispersed, forming 2 smaller sub-clusters: one composed of SA1 (Alcohol) and SA2 (Opioids), and the other of SA3 (Cocaine) and SA5 (Amphetamines). Both sub-clusters were situated on the periphery of the PANSS complex. SA4 (Cannabis) was situated between the CT and PANSS complexes, serving as a bridge between the 2 substance abuse sub-clusters.

Among the 5 CT nodes, only 2—CT1 (Sexual Abuse) and CT4 (Emotional Neglect)—showed direct connections to the broader network, suggesting that these 2 forms of CT play a particularly important role in linking trauma experiences with psychopathology and substance use.

Three distinct pathways connected trauma to other parts of the network:

1. CT1 (Sexual Abuse) → GP6 (Depression) → N4 (Passive/Apathetic Social Withdrawal), GP2 (Anxiety), GP3 (Guilt Feelings), GP7 (Motor Retardation).
2. CT4 (Emotional Neglect) → GP11 (Poor Attention) → P2 (Conceptual Disorganization), GP10 (Disorientation), GP15 (Preoccupation).
3. CT4 (Emotional Neglect) → SA4 (Cannabis) → N3 (Poor Rapport), GP3 (Guilt Feelings), GP4 (Tension), GP7 (Motor Retardation), GP12 (Lack of Judgment and Insight).

The primary gateways from the substance abuse domain to the rest of the network were SA2 (Opioids) and SA4 (Cannabis), both of which were linked to several negative and general psychopathology symptoms. In contrast, SA1 (Alcohol), SA3 (Cocaine), and SA5 (Amphetamines) only connected with other substance abuse nodes and showed no direct links to CT or PANSS nodes.

The network obtained from the secondary analysis using EBIC is presented in the Figure S1. Although this network was sparser than the main one, its overall clustering, structural organization, and the key bridges between CT nodes, substance abuse nodes, and PANSS nodes remained consistent with the primary network.

### Node Strength, Bridge Strength, and Predictability

**Figure 2:**
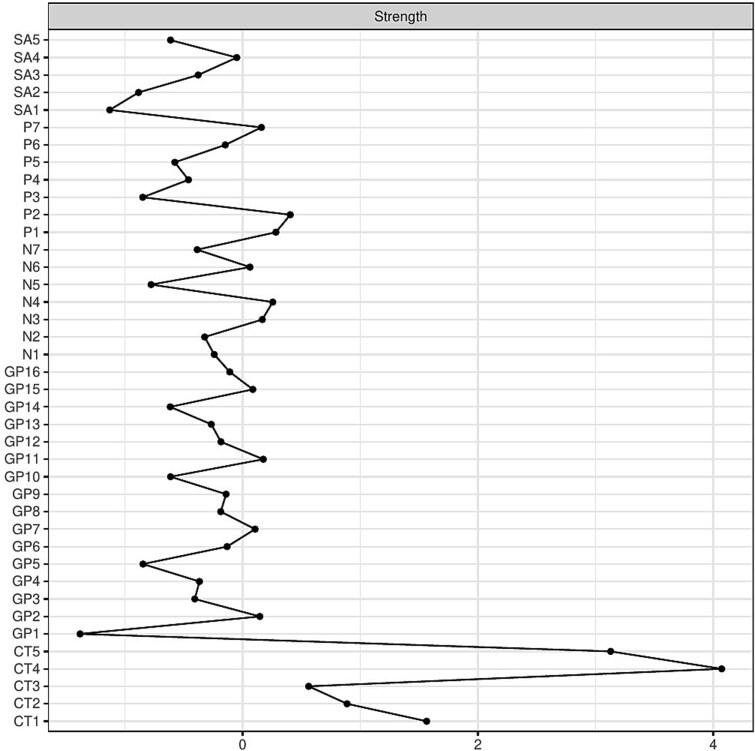
Line Plot Depicting Standardized Node Strength (z-Scores) for all Nodes in the Network. Nodes are arranged vertically along the y-axis, while their corresponding strength values are shown on the *x*-axis. CT1 = sexual abuse; CT2 = physical abuse; CT3 = emotional abuse; CT4 = emotional neglect; CT5 = physical neglect; SA1 = alcohol; SA2 = opioids; SA3 = cocaine; SA4 = cannabis; SA5 = amphetamines; P1 = delusions; P2 = conceptual disorganization; P3 = hallucinations; P4 = excitement; P5 = grandiosity; P6 = suspiciousness/persecution; P7 = hostility; N1 = blunted affect; N2 = emotional withdrawal; N3 = poor rapport; N4 = passive/apathetic social withdrawal; N5 = difficulty in abstract thinking; N6 = lack of spontaneity and flow of conversation; N7 = stereotyped thinking; GP1 = somatic concern; GP2 = anxiety; GP3 = guilt feelings; GP4 = tension; GP5 = mannerisms and posturing; GP6 = depression; GP7 = motor retardation; GP8 = uncooperativeness; GP9 = unusual thought content; GP10 = disorientation; GP11 = poor attention; GP12 = lack of judgment and insight; GP13 = disturbance of volition; GP14 = poor impulse control; GP15 = preoccupation; GP16 = active social avoidance.

The 5 CT nodes exhibited the highest node strength in the network, indicating strong overall connectivity with other variables. However, much of this strength reflected their robust interconnections within the CT cluster itself. CT4 (Emotional Neglect) had the highest node strength overall. Among PANSS items, the most central nodes (in descending order of node strength) were: P2 (Conceptual Disorganization), P1 (Delusions), N4 (Passive/Apathetic Social Withdrawal), N3 (Poor Rapport), GP11 (Poor Attention), P7 (Stereotyped Thinking), GP2 (Anxiety), GP7 (Motor Retardation), GP15 (Preoccupation), and N6 (Lack of Spontaneity and Flow of Conversation). Among substance abuse nodes, SA4 (Cannabis) was the most central. Node strength for all variables is depicted in Figure 2, with exact values provided in Table S2.

Bridge strength analyses (Table S3) revealed that CT4 (Emotional Neglect), SA4 (Cannabis), and GP11 (Poor Attention) had the highest bridge centrality within their respective communities, indicating that they serve as major connecting points between the domains of trauma, substance use, and psychopathology.

The mean predictability of the network was 0.42, indicating that, on average, 42% of the variance in each variable was explained by its connections with other nodes. This suggests a moderately predictable and well-integrated network structure. Predictability values for all nodes are presented in Table S4.

### Network Stability and Accuracy

The 95% bootstrapped confidence intervals indicated acceptable stability, with CI-to-weight ratios—calculated as the size of the confidence interval divided by the edge weight—less than or equal to 1 for 80 of the 93 edges (Table S1). Case-drop bootstrapping (Figure S2) showed that node strength estimates remained consistent with higher percentages of data retained and degraded only gradually with increasing case removal. A correlation stability coefficient (CS-coefficient) of 0.36, exceeding the 0.25 threshold for acceptable centrality accuracy,^36^ further supports the accuracy of node strength estimates. Together, these procedures provide evidence on the robustness of the network’s estimated structure and centrality metrics.

## Discussion

**Figure 3:**
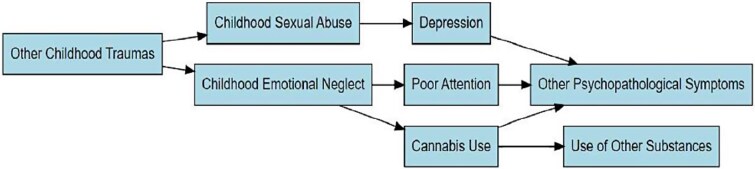
Flowchart Illustrating the Information Flow Within the Network, Depicting Patterns of Connection from Childhood Trauma Domains to Substance Use and Psychopathology.

To our knowledge, this is the first study to use network analysis to investigate the interplay between CT, substance use, and clinical symptoms in an early psychosis population. Three key findings emerged: (1) only 2 CT subtypes—sexual abuse and emotional neglect—showed direct links to the broader network, with emotional neglect emerging as the most central node; (2) cannabis use played a pivotal role in connecting different substances and bridging substance use with CT and psychopathology; (3) cannabis use, along with depression and poor attention, served as bridging nodes linking CT to psychopathology. These findings are illustrated in Figure 3. Notably, the key findings described above were consistent across both the primary network estimated via CV and the more conservative network estimated via EBIC, underscoring their robustness.

Among the CT subtypes, only sexual abuse and emotional neglect were directly connected to the rest of the network. In contrast, physical abuse, emotional abuse, and physical neglect were only associated with other CT subtypes, suggesting that their influence on the broader network may propagate through co-occurring forms of trauma. This pattern is consistent with previous network studies^15^^,^^16^ and provides further support for the notion that CT exerts a cumulative effect both on SUD^42^^,^^43^ and on psychopathology in early psychosis.^44^

Emotional neglect exhibited the highest node strength and bridge strength among CT nodes, indicating a prominent role in activating the broader network. This finding aligns with a network analysis study by Sideli et al.,^45^ which explored CT, substance use, and psychopathology in a general population sample. Emotional neglect may be particularly impactful in the context of multiple traumas, as the absence of attentive support could exacerbate the effects of more overtly violent forms of CT,^45^ thereby playing a critical role in the development of psychopathology.^46^^,^^47^

The 5 substances formed 2 distinct subclusters: one comprising sedative substances (alcohol and opioids), and the other comprising stimulants (cocaine and amphetamines). Cannabis occupied a dual role by bridging these 2 subclusters, as well as serving as a link between substance use, CT, and psychopathology. This dual role of cannabis is consistent with findings from Sideli et al.^45^

Cannabis is known to frequently co-occur with both sedative and stimulant substances.^48^ Although only current substance use was assessed in this study, if the observed pathway from CT to other substances via cannabis is interpreted within a causal framework, it supports the “gateway” hypothesis—the idea that cannabis may facilitate the use of other substances,^49^^,^^50^ particularly among individuals with psychiatric comorbidities.^50^

Three nodes—cannabis use, depression, and poor attention—emerged as key bridges linking CT to the psychopathology domain. Each was directly connected to several of the network’s most central symptoms, effectively functioning as hubs that facilitate the activation of clinical symptoms across the network.

The association between emotional neglect and cannabis use aligns with previous research identifying CT as a risk factor for cannabis use,^2^ possibly through a self-medication mechanism.^43^ Traumatic experiences may heighten negative affect, leading some individuals to use substances such as cannabis as a means of coping with or alleviating these distressing emotional states. The role of cannabis as a bridge between CT and psychopathology observed in this study mirrors findings from general population research indicating that cannabis use can mediate^11^^,^^12^ or amplify^6^^,^^7^ the relationship between CT and psychotic symptoms.

Depression is highly prevalent in individuals with PD who have experienced CT, often reflecting affect dysregulation, a known consequence of early trauma.^1^ Previous studies support the mediating role of depressive symptoms in the relationship between CT and psychosis,^51^ and other network studies have also found depression to be a key bridge node between trauma and psychopathology in PD.^15^^,^^16^

Poor attention, frequently observed in early psychosis,^52^ is part of the broader spectrum of neurocognitive impairments observed in PD.^53^ Our findings are consistent with evidence linking CT to attention deficits^54^ and suggesting that attention mediates the relationship between childhood neglect and several symptoms in PD.^55^

Accordingly, the 2 symptoms bridging CT and psychopathology—depression and poor attention—are associated with affect dysregulation and neurocognitive deficits, respectively. These 2 mechanisms correspond to the affective and cognitive pathways to psychosis,^56^ both of which have been linked to increased vulnerability to the disorder^56^^,^^57^—conceptualized as an individual’s enduring susceptibility to developing a psychotic disorder. The identification in this study of a third, cannabis-related pattern of connection, independently linking CT to psychopathology is particularly noteworthy.

Several studies^5^^,^^58^^,^^59^ have suggested that cannabis users who go on to develop PD may possess a lower vulnerability to psychosis than their non-using counterparts. In other words, when cannabis is conceptualized as a stressor within the classical stress–vulnerability framework, its presence may reduce the threshold of vulnerability required for the onset of psychosis.^5^

Building on this perspective, it could be hypothesized that, in this study, the connections via depression and poor attention might reflect patterns observed among individuals with higher vulnerability to psychosis. In contrast, the cannabis-related connection could represent a pattern more prominent among individuals with lower vulnerability.

This hypothesis may also help explain the discrepancy between our findings and those of the Bosma et al.^19^ study, which examined individuals with chronic psychotic disorders and their healthy siblings and found no bridging role for cannabis between CT and psychopathology in either group. Early psychosis cohorts often include individuals who may never progress to chronic psychosis and might represent a subgroup with intermediate levels of vulnerability—lower than that of chronic patients but higher than that of individuals without psychotic disorders. For these individuals, cannabis use might serve as a critical additional stressor that facilitates the transition to psychosis.

Of note, given the cross-sectional design of this study, alternative non-causal explanations should also be considered for the observed CT–cannabis use–psychopathology pattern. This association may, in fact, reflect a shared underlying vulnerability among these constructs. For example, insecure attachment has been proposed as a predisposing factor that increases the likelihood of experiencing CT, subsequent cannabis use,^60^ and greater severity of psychotic symptoms,^61^ which may partly account for the observed association.

### Limitations

The findings should be interpreted in light of several limitations. A key limitation of this study is its cross-sectional design. Networks derived from cross-sectional data can provide a useful roadmap of statistical predictive relationships between variables. These relationships can be interpreted as putative causal links and used to generate hypotheses about potential pathways. However, the network estimated in this study is undirected and therefore cannot determine the direction or temporal sequence of associations. Consequently, any inferences about causal effects remain speculative. Longitudinal research is required to establish the temporal direction of these associations and to uncover underlying causal pathways.

Another limitation relates to the low frequency of certain substance use categories, namely opioids, cocaine, and amphetamines. These variables were retained due to their clinical relevance. However, although the bootstrapped confidence intervals for the associated edges suggested acceptable stability within the studied sample, these results should still be interpreted with caution. Given the limited frequency of these substances, the observed stability may primarily reflect consistent patterns within this particular sample rather than reliable population-level associations. Future research with larger and more balanced samples of substance users is needed to validate and expand upon these findings.

To ensure sufficient statistical power and produce stable, interpretable results, we focused on the CT types that are reported as most prevalent in the literature. Future studies should extend this approach to include less frequent trauma types to capture a broader range of associations. Although the assessment of CT was conducted over a 3-year period and, when possible, corroborated with information from family members—thereby reducing potential bias—the retrospective nature of the assessment may still have introduced some recall bias.

Several other methodological factors may also have influenced the results. Variability in assessment timing (2 vs. 6 months), while enhancing representativeness by allowing inclusion of participants who were initially too symptomatic to complete the evaluation, may have introduced selection bias, as the timing of assessment was related to patients’ clinical stability. Furthermore, variation in medication use and adherence—with more than half of patients unmedicated and only a limited proportion of medicated individuals fully compliant—may have introduced confounding by treatment effects, as differences in symptom severity could partly reflect disparities in treatment exposure rather than true associations.

In this study, cannabis emerged as a key bridge linking CT to psychopathology. However, the relationship between cannabis use and PD is likely multifaceted and influenced by several moderating factors, including age at first use, use during adolescence, type and potency of cannabis used, as well as specific genetic polymorphisms.^62^ While examining these factors was beyond the scope of the current study, future research exploring the interplay between CT, cannabis use, and psychopathology should incorporate these dimensions to provide a more comprehensive understanding of the mechanisms underlying this association.

Finally, this study provides evidence for multiple patterns linking CT to psychopathology and advances the hypothesis that these distinct patterns may reflect differing levels of vulnerability to psychosis. However, vulnerability was inferred from the existing literature rather than measured using indicators of stress sensitivity or neurocognitive functioning. Future research should incorporate objective indices of vulnerability to psychosis—such as assessments of stress sensitivity, comprehensive neurocognitive assessments, or neuroimaging data—to validate and refine these proposed patterns.

## Conclusions

This study, employing network analysis in a transdiagnostic early psychosis sample, offers novel insights into the associations linking CT, substance use, and psychopathology in PD. The findings reveal distinct trauma-related patterns of connection to symptoms that may reflect varying levels of vulnerability to psychosis, providing a potential framework for understanding individual differences in how trauma shapes clinical outcomes. Cannabis emerged as uniquely positioned among substances, potentially acting as both a catalyst for the development of psychopathology and a gateway to the use of other substances in individuals with a history of trauma. These findings contribute to the growing evidence for the high granularity of trauma- and illness-related constructs and the complex patterns of association among them. Appreciating this complexity may help refine how these dimensions are conceptualized, ultimately supporting their more comprehensive and individualized measurement and integration into clinical care planning in early intervention settings.

## Supplementary Material

supplementary_material_sbaf253

## References

[1] Stanton KJ, Denietolis B, Goodwin BJ, Dvir Y. Childhood trauma and psychosis: an updated review. *Child Adolesc Psychiatr Clin*. 2020;29:115-129. 10.1016/j.chc.2019.08.004 31708041

[2] Cicchetti D, Handley ED. Child maltreatment and the development of substance use and disorder. *Neurobiol Stress*. 2019;10:100-144. 10.1016/j.ynstr.2018.100144 PMC6430405 30937350

[3] Moustafa AA, Parkes D, et al. The relationship between childhood trauma, early-life stress, and alcohol and drug use, abuse, and addiction: an integrative review. Curr Psychol. 2021;40:579-584. 10.1007/s12144-018-9973-9

[4] Khokhar JY, Dwiel LL, Henricks AM, Doucette WT, Green AI. The link between schizophrenia and substance use disorder: a unifying hypothesis. *Schizophr Res*. 2018;194:78-85. 10.1016/j.schres.2017.04.016 28416205 PMC6094954

[5] Løberg EM, Helle S, Nygård M, Berle JØ, Kroken RA, Johnsen E. The cannabis pathway to non-affective psychosis may reflect less neurobiological vulnerability. *Front Psychiatry*. 2014;5:159. 10.3389/fpsyt.2014.00159 25477825 PMC4235385

[6] Houston JE, Murphy J, Adamson G, Stringer M, Shevlin M. Childhood sexual abuse, early cannabis use, and psychosis: testing an interaction model based on the National Comorbidity Survey. *Schizophr Bull*. 2008;34:580-585. 10.1093/schbul/sbm127 18024467 PMC2632429

[7] Konings M, Stefanis N, Kuepper R, et al. Replication in two independent population-based samples that childhood maltreatment and cannabis use synergistically impact on psychosis risk. *Psychol Med*. 2012;42:149-159. 10.1017/S0033291711000973 21676285

[8] Ajnakina O, Borges S, Forti D, et al. Role of environmental confounding in the association between FKBP5 and first-episode psychosis. *Front Psychiatry*. 2014;5:84. 10.3389/fpsyt.2014.00084 25101008 PMC4101879

[9] Sideli L, Fisher HL, Murray RM, et al. Interaction between cannabis consumption and childhood abuse in psychotic disorders: preliminary findings on the role of different patterns of cannabis use. *Early Interv Psychiatry*. 2018;12:135-142. 10.1111/eip.12285 26560802

[10] van Nierop M, Van Os J, Gunther N, et al. Does social defeat mediate the association between childhood trauma and psychosis? Evidence from the NEMESIS-2 study. *Acta Psychiatr Scand*. 2014;29:467-476. 10.1111/acps.12212 24571736

[11] Whitfield CL, Dube SR, Felitti VJ, Anda RF. Adverse childhood experiences and hallucinations. *CABND3*. 2005;29:797-810. 16051353 10.1016/j.chiabu.2005.01.004

[12] Trotta G, Spinazzola E, Degen H, et al. The impact of childhood trauma and cannabis use on paranoia: a structural equation model approach. *Psychol Med*. 2025;55:e220. 40776588 10.1017/S0033291725101190 PMC12360688

[13] Borsboom D, Cramer AO. Network analysis: an integrative approach to the structure of psychopathology. *Annu Rev Clin Psychol*. 2013;9:91-121. 10.1146/annurev-clinpsy-050212-185608 23537483

[14] Alameda L, Christy A, Rodriguez V, et al. Association between specific childhood adversities and symptom dimensions in people with psychosis: systematic review and meta-analysis. *Schizophr Bull*. 2021;47:975-985. 10.1093/schbul/sbaa199 33836526 PMC8266673

[15] Isvoranu AM, van Borkulo CD, Boyette LL, et al. A network approach to psychosis: pathways between childhood trauma and psychotic symptoms. *Schizophr Bull*. 2017;43:187-196. 10.1093/schbul/sbw055 27165690 PMC5216845

[16] Kiakos D, Alameda L, Lepreux I, et al. Pathways between childhood trauma, clinical symptoms, and functioning in new-onset psychosis: novel insights from a network analysis approach. *Schizophrenia*. 2025;11:1-9. 40374630 10.1038/s41537-025-00620-2 PMC12081628

[17] Schlesselmann AJ, Huntjens RJ, Renard SB, et al. A network approach to trauma, dissociative symptoms, and psychosis symptoms in schizophrenia spectrum disorders. *Schizophr Bull*. 2023;49:559-568. 10.1093/schbul/sbac122 36124634 PMC10154708

[18] Foti DJ, Kotov R, Guey LT, Bromet EJ. Cannabis use and the course of schizophrenia: 10-year follow-up after first hospitalization. *Am J Psychiatry*. 2010;167:987-993. 10.1176/appi.ajp.2010.09020189 20478874 PMC3594105

[19] Bosma MJ, Marsman M, Vermeulen JM, et al. Exploring the interactions between psychotic symptoms, cognition, and environmental risk factors: a Bayesian analysis of networks. *Schizophr Bull*. 2024;51:1134-1145. 10.1093/schbul/sbae174 PMC12236352 39401320

[20] Baumann PS, Crespi S, Marion-Veyron R, et al. Treatment and early intervention in psychosis program (TIPP-Lausanne): implementation of an early intervention program for psychosis in Switzerland. *Early Interv Psychiatry*. 2013;7:322-328. 10.1111/eip.12037 23445318

[21] Yung AR, Yuen HP, McGorry PD, et al. Mapping the onset of psychosis: the comprehensive assessment of At-risk mental states. *Aust N Z J Psychiatry*. 2005;39:964-971. 10.1080/j.1440-1614.2005.01714.x 16343296

[22] Bernstein DP, Stein JA, Newcomb MD, et al. Development and validation of a brief screening version of the childhood trauma questionnaire. *CABND3*. 2003;27:169-190. 12615092 10.1016/s0145-2134(02)00541-0

[23] Van Nierop M, Lataster T, Smeets F, et al. Psychopathological mechanisms linking childhood traumatic experiences to risk of psychotic symptoms: analysis of a large, representative population-based sample. *Schizophr Bull*. 2014;40:S123-S130. 10.1093/schbul/sbt150 24562491 PMC3934395

[24] Drake RE, Osher FC, Wallach MA. Alcohol use and abuse in schizophrenia: a prospective community study. *J Nerv Ment Dis*. 1989;177:408-414. 10.1097/00005053-198907000-00004 2746194

[25] Drake RE, Wallach MA. Substance abuse among the chronic mentally ill. *Psychiatr Serv*. 1989;40:1041-1046. 10.1176/ps.40.10.1041 2807205

[26] Hunt GE, Large MM, Cleary M, Lai HMX, Saunders JB. Prevalence of comorbid substance use in schizophrenia spectrum disorders in community and clinical settings, 1990–2017: systematic review and meta-analysis. *Drug Alcohol Depend*. 2018;191:234-258. 10.1016/j.drugalcdep.2018.07.011 30153606

[27] Kay SR, Fiszbein A, Opler LA. The positive and negative syndrome scale (PANSS) for schizophrenia. *Schizophr Bull*. 1987;13:261-276. 10.1093/schbul/13.2.261 3616518

[28] Geck S, Roithmeier M, Bühner M, et al. COSMIN systematic review and meta-analysis of the measurement properties of the positive and negative syndrome scale (PANSS). *EClinicalMedicine*. 2025;82:103-155. 10.1016/j.eclinm.2025.103155 PMC12008685 40255437

[29] Haslbeck JM, Waldorp LJ. MGM: estimating time-varying mixed graphical models in high-dimensional data. *J Stat Softw*. 2020;93:1-46.

[30] Blanken TF, Isvoranu AM, Epskamp S. Estimating network structures using model selection. In: Isvoranu AM, Epskamp S, Waldorp LJ, Borsboom D (eds.), *Network Psychometrics with R*. Routledge, 2022.

[31] Fried EI, von Stockert S, Haslbeck JMB, Lamers F, Schoevers RA, Penninx BWJH. Using network analysis to examine links between individual depressive symptoms, inflammatory markers, and covariates. *Psychol Med*. 2020;50:2682-2690. 10.1017/S0033291719002770 31615595

[32] Gawęda Ł, Pionke R, Hartmann J, Nelson B, Cechnicki A, Frydecka D. Toward a complex network of risks for psychosis: combining trauma, cognitive biases, depression, and psychotic-like experiences on a large sample of young adults. *Schizophr Bull*. 2021;47:395-404. 10.1093/schbul/sbaa125 33728467 PMC7965064

[33] Liu H, Lafferty J, Wasserman L. The nonparanormal: semiparametric estimation of high dimensional undirected graphs. *J Mach Learn Res*. 2009;10:2295-2328. PMC4729207 26834510

[34] Costantini G, Epskamp S, Borsboom D, et al. State of the aRt personality research: a tutorial on network analysis of personality data in R. *J Res Pers*. 2015;2015:13-29.

[35] Isvoranu AM, Epskamp S. Which estimation method to choose in network psychometrics? Deriving guidelines for applied researchers. *Psychol Methods*. 2023;28:925. 10.1037/met0000439 34843277

[36] Deserno MK, Isvoranu AM, Epskamp S, Blanken TF. Descriptive analyses of network structures. In: Isvoranu AM, Epskamp S, Waldorp LJ, Borsboom D (eds.), *Network Psychometrics with R*. Routledge, 2022.

[37] Epskamp S, Borsboom D, Fried EI. Estimating psychological networks and their accuracy: a tutorial paper. *Behav Res Methods*. 2018;50:195-212. 10.3758/s13428-017-0862-1 28342071 PMC5809547

[38] Jones PJ, Ma R, McNally RJ. Bridge centrality: a network approach to understanding comorbidity. *Multivar Behav Res*. 2021;56:353-367. 10.1080/00273171.2019.1614898 31179765

[39] Haslbeck JM, Waldorp LJ. How well do network models predict observations? On the importance of predictability in network models. *Behav Res Methods*. 2018;50:853-861. 10.3758/s13428-017-0910-x 28718088 PMC5880858

[40] R. Core Team . R: A Language and Environment for Statistical Computing. R Core Team, 2013.

[41] Epskamp S, Cramer AO, Waldorp LJ, Schmittmann VD, Borsboom D. Qgraph: network visualizations of relationships in psychometric data. *J Stat Softw*. 2012;48:1-18.

[42] Hoffmann JP, Jones MS. Cumulative stressors and adolescent substance use: a review of 21st-century literature. *TVA*. 2022;23:891-905. 33345723 10.1177/1524838020979674

[43] Leza L, Siria S, López-Goñi JJ, Fernandez-Montalvo J. Adverse childhood experiences (ACEs) and substance use disorder (SUD): a scoping review. *Drug Alcohol Depend*. 2021;221:108563. 10.1016/j.drugalcdep.2021.108563 33561668

[44] Trauelsen AM, Bendall S, Jansen JE, et al. Childhood adversity specificity and dose-response effect in non-affective first-episode psychosis. *Schizophr Res*. 2015;165:52-59. 10.1016/j.schres.2015.03.014 25868932

[45] Sideli L, Lo Coco G, Albano A, et al. Substance addictive behaviors and their relationship with interpersonal trauma, emotion dysregulation, and psychopathological symptoms: a correlation network approach. *Int J Ment Health Addict*. 2025;23:964-982.

[46] Schimmenti A . The trauma factor: examining the relationships among different types of trauma, dissociation, and psychopathology. *J Trauma Dissociation*. 2018;19:552-571. 29125800 10.1080/15299732.2017.1402400

[47] Schimmenti A, Bifulco A. Linking lack of care in childhood to anxiety disorders in emerging adulthood: the role of attachment styles. *Child Adolesc Ment Health*. 2015;20:41-48. 10.1111/camh.12051 32680332

[48] Hasin D, Walsh C. Cannabis use, cannabis use disorder, and comorbid psychiatric illness: a narrative review. *J Clin Med*. 2020;10:15. 33374666 10.3390/jcm10010015 PMC7793504

[49] Silins E, Horwood LJ, Patton GC, et al. Young adult sequelae of adolescent cannabis use: an integrative analysis. *Lancet Psychiatry*. 2014;1:286-293. 10.1016/S2215-0366(14)70307-4 26360862

[50] Secades-Villa R, Garcia-Rodriguez O, Jin CJ, Wang S, Blanco C. Probability and predictors of the cannabis gateway effect: a national study. *Int J Drug Policy*. 2015;26:135-142. 10.1016/j.drugpo.2014.07.011 25168081 PMC4291295

[51] Sideli L, Murray RM, Schimmenti A, et al. Childhood adversity and psychosis: a systematic review of bio-psycho-social mediators and moderators. *Psychol Med*. 2020;50:1761-1782. 10.1017/S0033291720002172 32624020

[52] Egeland J . Frequency of attention deficit in first-episode schizophrenia compared to ADHD. *Appl Neuropsychol*. 2010;17:125-134. 10.1080/09084280903297859 20467954

[53] de Gracia Dominguez M, Viechtbauer W, Simons CJ, van Os J, Krabbendam L. Are psychotic psychopathology and neurocognition orthogonal? A systematic review of their associations. *Psychol Bull*. 2009;135:157. 10.1037/a0014415 19210058

[54] Vargas T, Lam PH, Azis M, Osborne KJ, Lieberman A, Mittal VA. Childhood trauma and neurocognition in adults with psychotic disorders: a systematic review and meta-analysis. *Schizophr Bull*. 2019;45:1195-1208. 10.1093/schbul/sby150 30376115 PMC6811825

[55] Mansueto G, Schruers K, Cosci F, et al. Childhood adversities and psychotic symptoms: the potential mediating or moderating role of neurocognition and social cognition. *Schizophr Res*. 2019;206:183-193. 10.1016/j.schres.2018.11.028 30527930

[56] Myin-Germeys I, van Os J. Stress-reactivity in psychosis: evidence for an affective pathway to psychosis. *Clin Psychol Rev*. 2007;27:409-424. 10.1016/j.cpr.2006.09.005 17222489

[57] Harvey PD, McClure MM, Patterson TL, et al. Impairment in functional capacity as an endophenotype candidate in severe mental illness. *Schizophr Bull*. 2012;38:1318-1326. 10.1093/schbul/sbr046 21562142 PMC3494058

[58] Sabe M, Zhao N, Kaiser S. Cannabis, nicotine and the negative symptoms of schizophrenia: systematic review and meta-analysis of observational studies. *Neurosci Biobehav Rev*. 2020;116:415-425. 10.1016/j.neubiorev.2020.07.007 32679232

[59] Burns JK . Pathways from cannabis to psychosis: a review of the evidence. *Front Psychiatry*. 2013;4:128. 10.3389/fpsyt.2013.00128 24133460 PMC3796266

[60] Carley S, Adams GC. The interaction between attachment, trauma and cannabis in creating vulnerability for psychosis. *Psychosis*. 2024;16:207-211. 10.1080/17522439.2023.2177326

[61] Puckett J, Sood M, Newman-Taylor K. Does insecure attachment lead to psychosis via dissociation? A systematic review of the literature. *PAPTRAP*. 2024;97:372-392. 10.1111/papt.12521 38358073

[62] Groening JM, Denton E, Parvaiz R, et al. A systematic evidence map of the association between cannabis use and psychosis-related outcomes across the psychosis continuum: an umbrella review of systematic reviews and meta-analyses. *Psychiatry Res*. 2024;331:115626. 10.1016/j.psychres.2023.115626 38096722

