# Supplementary material for: Mapping the Interplay Between Childhood Trauma, Substance Use, and Psychopathology in Early Psychosis: A Network Analysis Approach
Source: Schizophr Bull. 2026 Mar 21;52(2):sbaf253. doi: 10.1093/schbul/sbaf253 (PMC13005114; doi:10.1093/schbul/sbaf253)
Supplement: supplementary_material_sbaf253 [file supplementary_material_sbaf253.zip › supplementary_material_sbaf253.rtf]

Mapping the Interplay between Childhood Trauma, Substance Use, and Psychopathology in Early Psychosis: A Network Analysis Approach

SUPPLEMENTARY MATERIAL

Supplementary Note 1.

Case Manager Rating Scale

Instructions: Assess the severity of substance use by your patient over the past month using the scale below. Focus on the period of most significant concern during this time. To complete this evaluation, consider information provided by the patient during interviews, behavioral observations, and collateral reports from family, day centers, and the community, among others.

Mark the substances used:
Alcohol, Cannabis, Cocaine, Hallucinogens, Opioids, PCP, Amphetamines, Sedatives/Hypnotics/Anxiolytics, Non-prescribed Medications, Other.
Assign a score (1–5) for each substance used.

1	Absent	The patient has not used the substance during this period.
	
2	Mild	The patient has used the substance during this period, but there are no signs of persistent or recurrent issues in social, occupational, psychological, or physical functioning related to substance use, nor is there any evidence of dangerous use.
	
3	Moderate	The patient has used the substance during this period, and there are indications of persistent or recurrent problems in social, occupational, psychological, or physical functioning associated with substance use, or evidence of recurrent dangerous use. These issues have persisted for at least one month. For example, recurring substance use has led to disruptive behaviors and housing difficulties.
	
4	Severe	Meets the criteria for "moderate" use, plus at least three of the following: consumption in larger quantities or for a longer duration than intended, significant time spent obtaining or using the substance, important activities are abandoned due to substance use, continued use despite awareness of substance-related problems, evident tolerance, withdrawal symptoms, or using the substance to alleviate or prevent withdrawal symptoms. For example, binge drinking and excessive preoccupation with alcohol have caused the patient to forgo vocational training and social activities where substance use is not present.
	
5	Extremely Severe	Meets the criteria for "severe" use, with substance-related problems so severe that they impair the patient's ability to function outside of an institutional setting. For instance, persistent substance use leads to disruptive behaviors and an inability to pay rent, resulting in frequent police involvement and a need for hospitalization.	


Supplementary Table 1.

List of network edges with weights, nonparametric bootstrap 2.5th and 97.5th quantiles, and corresponding 95% confidence intervals.

Edge	Weight	Bootstrapped 2.5th quantile estimate	Bootstrapped 97.5th quantile estimate	95 % bootstrapped confidence interval	Confidence Interval to Weight Ratio	
CT1-CT2	0.62	0.50	0.74	0.24	0.39	
CT1-CT4	0.76	0.36	1.16	0.80	1.05	
CT1-CT5	0.52	0.25	0.79	0.54	1.04	
CT1-GP6	0.18	0.13	0.23	0.10	0.56	
CT2-CT3	0.99	0.88	1.10	0.22	0.22	
CT3-CT5	0.40	0.27	0.53	0.26	0.65	
CT4-CT5	2.24	1.50	2.98	1.48	0.66	
CT4-SA4	0.25	0.17	0.32	0.15	0.60	
CT4-GP11	0.56	0.30	0.80	0.50	0.89	
SA1-SA2	0.23	0.17	0.28	0.11	0.48	
SA2-SA4	0.11	0.09	0.12	0.03	0.27	
SA2-GP1	0.06	0.05	0.07	0.02	0.33	
SA3-SA4	0.16	0.14	0.17	0.03	0.19	
SA3-SA5	0.59	0.31	0.86	0.55	0.93	
SA4-N3	0.11	0.04	0.17	0.13	1.18	
SA4-GP3	0.10	0.09	0.11	0.02	0.20	
SA4-GP4	0.09	0.06	0.11	0.05	0.56	
SA4-GP7	0.10	0.05	0.14	0.09	0.90	
SA4-GP12	0.08	0.06	0.09	0.03	0.38	
P1-P3	0.19	0.13	0.24	0.11	0.58	
P1-P5	0.11	0.05	0.16	0.11	1.00	
P1-P6	0.30	0.14	0.45	0.31	1.03	
P1-GP3	0.10	0.09	0.11	0.02	0.20	
P1-GP9	0.50	0.22	0.77	0.55	1.10	
P2-P4	0.09	0.04	0.13	0.09	1.00	
P2-P5	0.08	0.04	0.11	0.07	0.88	
P2-N4	0.10	0.08	0.11	0.03	0.30	
P2-N5	0.17	0.11	0.22	0.11	0.65	
P2-N7	0.11	0.09	0.12	0.03	0.27	
P2-GP5	0.11	0.05	0.16	0.11	1.00	
P2-GP10	0.15	0.12	0.17	0.05	0.33	
P2-GP11	0.20	0.14	0.25	0.11	0.55	
P2-GP12	0.06	0.03	0.08	0.05	0.83	
P2-GP13	0.23	0.18	0.27	0.09	0.39	
P3-P5	0.07	0.03	0.10	0.07	1.00	
P3-GP9	0.10	0.08	0.11	0.03	0.30	
P3-GP10	0.07	0.04	0.09	0.05	0.71	
P4-P5	0.09	0.05	0.12	0.07	0.78	
P4-P7	0.21	0.12	0.29	0.17	0.81	
P4-N3	0.06	0.04	0.07	0.03	0.50	
P4-GP4	0.08	0.06	0.09	0.03	0.38	
P4-GP7	0.10	0.05	0.14	0.09	0.90	
P4-GP14	0.08	0.04	0.11	0.07	0.88	
P5-P7	0.13	0.08	0.17	0.09	0.69	
P5-GP11	0.05	0.04	0.05	0.01	0.20	
P5-GP9	0.11	0.07	0.14	0.07	0.64	
P6-N7	0.08	0.05	0.10	0.05	0.63	
P6-GP8	0.16	0.08	0.23	0.15	0.94	
P6-GP16	0.36	0.18	0.53	0.35	0.97	
P7-N7	0.12	0.09	0.14	0.05	0.42	
P7-GP8	0.15	0.09	0.20	0.11	0.73	
P7-GP14	0.50	0.21	0.78	0.57	1.14	
N1-N2	0.37	0.15	0.58	0.43	1.16	
N1-N6	0.11	0.06	0.15	0.09	0.82	
N1-GP7	0.36	0.26	0.45	0.19	0.53	
N2-N3	0.14	0.13	0.14	0.01	0.07	
N2-N4	0.28	0.15	0.40	0.25	0.89	
N3-N6	0.35	0.24	0.45	0.21	0.60	
N3-GP8	0.21	0.13	0.28	0.15	0.71	
N3-GP12	0.07	0.04	0.09	0.05	0.71	
N3-GP15	0.17	0.07	0.26	0.19	1.12	
N4-N5	0.09	0.04	0.13	0.09	1.00	
N4-GP6	0.21	0.19	0.22	0.03	0.14	
N4-GP13	0.09	0.06	0.11	0.05	0.56	
N4-GP16	0.41	0.27	0.54	0.27	0.66	
N5-GP8	0.10	0.08	0.11	0.03	0.30	
N5-GP10	0.11	0.09	0.12	0.03	0.27	
N6-GP3	0.11	0.10	0.11	0.01	0.09	
N6-GP7	0.30	0.24	0.35	0.11	0.37	
N6-GP13	0.07	0.04	0.09	0.05	0.71	
N6-GP15	0.11	0.06	0.15	0.11	1.00	
N7-GP12	0.15	0.06	0.23	0.17	1.13	
N7-GP15	0.27	0.21	0.32	0.11	0.41	
GP2-GP3	0.13	0.06	0.19	0.13	1.00	
GP2-GP4	0.38	0.17	0.58	0.41	1.08	
GP2-GP6	0.24	0.16	0.31	0.15	0.63	
GP2-GP12	0.11	0.05	0.16	0.11	1.00	
GP2-GP15	0.08	0.05	0.10	0.05	0.63	
GP2-GP16	0.16	0.12	0.19	0.07	0.44	
GP3-GP4	0.07	0.04	0.09	0.05	0.71	
GP3-GP5	0.05	0.03	0.06	0.03	0.60	
GP3-GP6	0.17	0.08	0.25	0.17	1.00	
GP4-GP8	0.06	0.02	0.09	0.07	1.17	
GP4-GP13	0.07	0.05	0.08	0.03	0.43	
GP5-GP13	0.11	0.06	0.15	0.09	0.82	
GP5-GP15	0.15	0.06	0.23	0.17	1.13	
GP6-GP7	0.12	0.05	0.18	0.13	1.08	
GP7-GP13	0.14	0.09	0.18	0.09	0.64	
GP9-GP12	0.20	0.13	0.26	0.13	0.65	
GP10-GP11	0.21	0.15	0.26	0.11	0.52	
GP10-GP12	0.05	0.04	0.05	0.01	0.20	
GP11-GP15	0.16	0.10	0.21	0.11	0.69	
GP13-GP15	0.12	0.06	0.17	0.11	0.92	


Supplementary Figure 1.
Partial correlation network estimated in the secondary analysis using EBIC (ã = 0.25)


Supplementary Table 2.

Absolute strength values of all network nodes

Node	Strength	
CT1	2.08	
CT2	1.61	
CT3	1.39	
CT4	3.80	
CT5	3.15	
SD1	0.23	
SD2	0.39	
SD3	0.74	
SD4	0.97	
SD5	0.58	
P1	1.20	
P2	1.28	
P3	0.42	
P4	0.69	
P5	0.61	
P6	0.90	
P7	1.11	
N1	0.84	
N2	0.78	
N3	1.12	
N4	1.18	
N5	0.47	
N6	1.05	
N7	0.74	
GP1	0.05	
GP2	1.10	
GP3	0.72	
GP4	0.75	
GP5	0.42	
GP6	0.91	
GP7	1.07	
GP8	0.87	
GP9	0.91	
GP10	0.58	
GP11	1.12	
GP12	0.88	
GP13	0.82	
GP14	0.58	
GP15	1.06	
GP16	0.93	


Supplementary Table 3. 

Absolute bridge strength values for network bridging nodes

Node	Bridge Strength	
CT1	0.18	
CT4	0.81	
SA2	0.05	
SA4	0.70	
N3	0.11	
GP1	0.05	
GP3	0.09	
GP4	0.09	
GP6	0.17	
GP7	0.06	
GP11	0.56	
GP12	0.08	


Supplementary Table 4. 

Predictability of the network's nodes

Node	Predictability	
CT1	0.17	
CT2	0.16	
CT3	0.00	
CT4	0.72	
CT5	0.36	
SD1	0.09	
SD2	0.16	
SD3	0.49	
SD4	0.30	
SD5	0.48	
P1	0.64	
P2	0.50	
P3	0.37	
P4	0.34	
P5	0.38	
P6	0.57	
P7	0.56	
N1	0.64	
N2	0.59	
N3	0.54	
N4	0.64	
N5	0.26	
N6	0.62	
N7	0.44	
GP1	0.11	
GP2	0.51	
GP3	0.25	
GP4	0.44	
GP5	0.33	
GP6	0.43	
GP7	0.61	
GP8	0.34	
GP9	0.59	
GP10	0.29	
GP11	0.38	
GP12	0.44	
GP13	0.37	
GP14	0.46	
GP15	0.48	
GP16	0.60	


Supplementary Figure 2. Average correlations between node strengths of networks with individuals removed and the original network. The line shows the mean correlation; the shaded area spans the 2.5% to 97.5% bootstrap quantiles.
